# Nationwide cohort analysis of pediatric urolithiasis: long-term metabolic, renal, and cardiovascular outcomes

**DOI:** 10.1007/s00467-026-07208-7

**Published:** 2026-03-09

**Authors:** Hsiao-Hui Yang, Wan-Ting Huang, Jen-Hung Wang, Je-Wen Liou, Hao-Jen Hsu, Ming-Chun Chen

**Affiliations:** 1 Department of Surgery, Hualien Tzu Chi Hospital, Buddhist Tzu Chi Medical Foundation, Hualien, 97004 Taiwan; 2 School of Medicine, Tzu Chi University, Hualien, 97004 Taiwan; 3 Institute of Medical Science, Tzu Chi University, Hualien, 97004 Taiwan; 4 Epidemiology and Biostatistics Center, Hualien Tzu Chi Hospital, Buddhist Tzu Chi Medical Foundation, Hualien, 97004 Taiwan; 5 Department of Biochemistry, School of Medicine, Tzu Chi University, Hualien, 97004 Taiwan; 6 Department of Biomedical Sciences and Engineering, Tzu Chi University, Hualien, 97004 Taiwan; 7 Department of Pediatrics, Hualien Tzu Chi Hospital, Buddhist Tzu Chi Medical Foundation, Hualien, 97004 Taiwan

**Keywords:** Pediatric urolithiasis, Metabolic risk, Chronic kidney disease, Cardiovascular complications, Nationwide cohort

## Abstract

**Background:**

Pediatric urolithiasis is increasingly recognized not merely as a localized urinary disorder but as part of a systemic metabolic–inflammatory process that may predispose to chronic kidney and cardiovascular disease. However, population-based data in Asian pediatric populations with urolithiasis remain limited. This nationwide study investigated the epidemiologic trends, metabolic risk factors, and systemic outcomes associated with pediatric urolithiasis in Taiwan.

**Methods:**

Using the National Health Insurance Research Database, children newly diagnosed with urolithiasis between January 2009 and December 2019 were identified and matched with controls according to age, sex, and index year. Comorbidities and medication exposures were evaluated, and outcomes including renal, cardiovascular, and metabolic complications were analyzed using Cox proportional hazards models.

**Results:**

Among 10,113 affected children and 101,130 matched controls, the annual incidence of pediatric urolithiasis declined from 23.85 to 16.22 per 100,000 persons. A male predominance and peak incidence during adolescence were noted. Major associated factors included hypercalciuria (adjusted hazard ratio [aHR] 31.54, with wide confidence intervals), congenital urinary anomalies (aHR 22.58), urinary tract infection (aHR 8.28), and exposure to diuretics or antibiotics. Compared with controls, children with urolithiasis had significantly higher risks of chronic kidney disease (aHR 5.92), hypertension (aHR 1.92), ischemic heart disease (aHR 1.93), and dyslipidemia (aHR 1.83).

**Conclusions:**

Despite a modest decline in incidence, pediatric urolithiasis remains a clinically important condition that is associated with an increased risk of long-term renal and cardiovascular morbidity. These findings highlight the importance of early metabolic evaluation, careful medication stewardship, and continued nephrology follow-up to mitigate chronic sequelae.

**Graphical abstract:**

**Figure:**
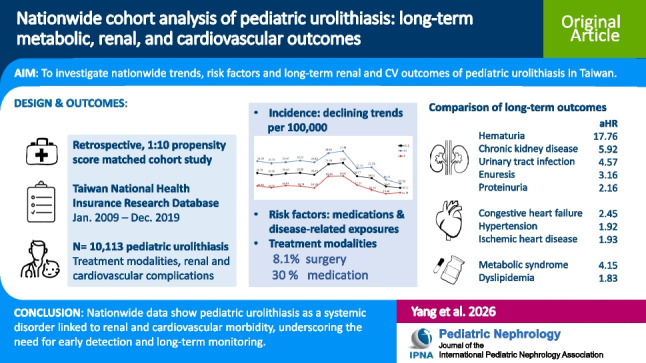
A higher resolution version of the Graphical abstract is available as Supplementary information Supplementary information

**Supplementary Information:**

The online version contains supplementary material available at 10.1007/s00467-026-07208-7.

## Introduction

Pediatric urolithiasis, which was previously considered a localized urinary disorder, is now increasingly recognized as part of a systemic process characterized by metabolic dysregulation and chronic low-grade inflammation [1, 2]. Recent adult studies have shown associations between stone disease, hypertension, dyslipidemia, insulin resistance, and endothelial dysfunction, indicating shared pathogenic pathways between nephrolithiasis and cardiovascular–renal injury [1, 3, 4]. However, such systemic associations remain underexplored in children, in whom early metabolic disturbances may have long-term consequences for renal and vascular health.

Globally, the incidence of pediatric urolithiasis has increased, reflecting changes in dietary habits, sedentary behavior, and climate [5]. Metabolic disorders account for 33–95% of cases. Meanwhile, anatomic malformations and infections contribute to 8–32% and 2–24%, respectively [5]. Despite advancements in metabolic screening and infection control, occult nephrolithiasis may still present with recurrent symptoms and abnormal urinary solutes even in the absence of imaging evidence [6]. Beyond morbidity and recurrence, stone disease has emerged as a systemic condition associated with renal and cardiovascular complications in both adults and adolescents [7, 8].

Previous population-based studies from Taiwan reported a 0.038% incidence of pediatric urolithiasis in 2007, with a peak occurrence during adolescence and a declining trend in incidence and healthcare utilization over time**.** Moreover, male patients exhibited an increasing frequency of upper urinary tract stone visits and higher recurrence rates (6.12% at 1 year and 34.71% at 5 years) [9, 10]. Nevertheless, comprehensive data on nationwide trends, predisposing factors, and long-term systemic outcomes in pediatric populations remain lacking. Using the National Health Insurance Research Database (NHIRD), which covers nearly the whole Taiwanese population, the current study aimed to investigate the epidemiology, metabolic and structural risk factors, and systemic complications—spanning renal, cardiovascular, and metabolic domains—of pediatric urolithiasis.

## Materials and methods

### Study design and data sources

This population-based retrospective cohort study used data from the Taiwan NHIRD, which includes medical information on more than 23 million residents. Patient records from January 2009 to December 2019 were obtained via the Health and Welfare Data Science Center [11, 12]. Urolithiasis cases were identified using diagnostic codes from both the *International Classification of Diseases, Ninth Revision, Clinical Modification* (ICD-9-CM, 2009–2015) and *Tenth Revision* (ICD-10-CM, 2016 onward). Annual incidence rates were calculated using population data from the Ministry of the Interior’s Public Birth Registry.

### Ethical approval and study population

The current study was conducted in accordance with the Declaration of Helsinki and approved by the Institutional Review Board of Hualien Tzu Chi Hospital (IRB111-023-B, March 28, 2022). The need for informed consent was waived owing to the retrospective nature of the study.

The pediatric urolithiasis cohort consisted of patients with a primary diagnosis of urinary tract calculi (ICD-9-CM 592, 594, 274.11, and 788.0; ICD-10-CM N13.2, N20.0–N20.2, N21, and N23) documented in at least one hospitalization or two outpatient visits. The first date of a qualifying diagnosis was defined as the index date [11]. Importantly, diagnostic codes indicating nephrocalcinosis were not included in the case definition to avoid misclassification with this distinct metabolic and radiologic entity. Hypercalciuria-related metabolic codes (e.g., ICD-9-CM 275.49; ICD-10-CM E83.59) were captured only as disease-related risk factors and not for defining urolithiasis. Patients with invalid or missing demographic data were excluded.

Children with pre-index predisposing factors were categorized into the disease- or medication-related groups. Medication exposure was assessed using the Anatomical Therapeutic Chemical (ATC) Classification System, focusing on prolonged use (> 30 days) of potential causative drugs. Patients with pre-existing comorbidities were included in the baseline analyses but excluded from the assessment of complication outcomes. Controls were randomly selected from the remaining NHIRD population and matched at a ratio of 1:10 according to sex, age, and index year (2009–2019), using identical exclusion criteria.

### Assessment of urolithiasis-related events and systemic complications

The outcomes associated with renal, cardiovascular, and metabolic systems were evaluated in both the urolithiasis and control groups [7, 8]. Systemic complications (e.g., chronic kidney disease, hypertension, dyslipidemia, metabolic syndrome, and ischemic heart disease) were identified using at least one hospitalization or two outpatient visits with the corresponding ICD-9/ICD-10 codes occurring ≥ 30 days after the index date. In addition, stone-related clinical events—such as hematuria, renal colic, urinary tract obstruction, and stone-related procedures—were captured separately as incident events occurring after the index date. These stone-related manifestations were not considered systemic long-term complications. Supplementary Table S1 lists all diagnostic, drug, and surgical codes used in this study.

### Evaluation of treatment modalities

The treatment strategies, including surgical and pharmacologic interventions, were identified using the ICD-9/ICD-10 procedure codes and ATC drug classifications. The surgical procedures included endoscopic cystolitholapaxy (78024C, 78026 C, and 78027 C), ureteroscopy with stone removal (77026B, 77027B, and 77028B), ureterolithotomy (77001B, 77002B, and 77030B), percutaneous nephrostolithotomy (76016B, 76017B), and cystolithotomy (78005B) [13].

The pharmacologic treatments were identified using ATC codes for agents commonly prescribed for urolithiasis [14, 15]:

- Diuretics: thiazides (C03A)
- Alpha-adrenergic blockers: G04CA01–G04CA04
- Smooth muscle relaxants: *hyoscine butylbromide* (Buscopan®, A03BB01) and *pipoxolan* (*Rowapraxin®*, G04BD)
- Uric acid-lowering agents: *allopurinol* (M04AA01), *benzbromarone* (M04AB03)
- Urinary alkalinizing agents**:** *potassium citrate* (A12BA02)

Patients who had used these medications for > 30 days prior to the index date were excluded to prevent confounding by previous treatment. Only patients who were newly prescribed with medications for > 30 days after diagnosis were included in the analysis [11].

### Statistical analysis

The annual incidence rates stratified by age and sex were calculated using population data from the Ministry of the Interior. Descriptive statistics were presented as counts and percentages. The incidence rates were compared using the chi-square test. Logistic regression models were used to estimate odds ratios (ORs) and adjusted odds ratios (aORs) for baseline risk factors of urolithiasis, whereas Cox proportional hazards models were applied to estimate adjusted hazard ratios (aHRs) for longitudinal outcomes. Incidence rates (IRs) and incidence rate ratios (IRRs) were calculated for event comparisons between groups. All analyses were conducted using SAS version 9.4 (SAS Institute Inc., Cary, NC, the USA). A two-tailed P value of < 0.05 was considered statistically significant.

## Results

### Demographic characteristics of the participants

**Fig. 1:**
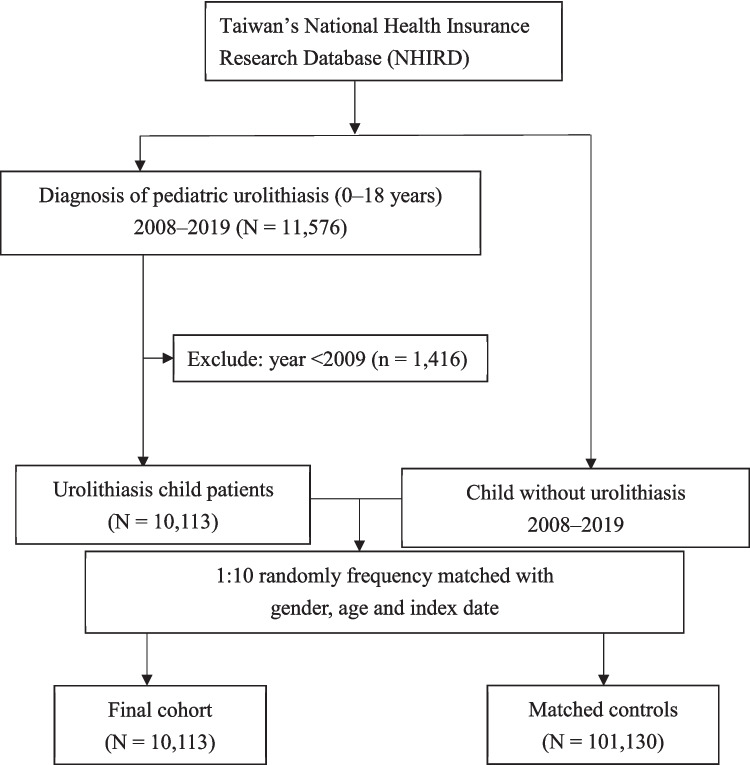
Selection of cohort with pediatric urolithiasis from the National Health Insurance Research Database (NHIRD). Flowchart illustrating the selection of pediatric patients with urolithiasis and matched controls from Taiwan’s National Health Insurance Research Database (NHIRD). In total, 11,576 patients aged 0–18 years who were diagnosed with urolithiasis between 2008 and 2019 were identified. After excluding 1,416 patients diagnosed before 2009 to ensure at least 1-year follow-up, 10,113 children with newly diagnosed urolithiasis between 2009 and 2018 were included in the final cohort. For each case, 10 control participants without a urolithiasis diagnosis were frequency-matched by age, sex, and index year, resulting in a comparison group of 101,130 individuals

From 2008 to 2019, 11,576 pediatric patients were diagnosed with urolithiasis in Taiwan (Fig. 1). After excluding patients diagnosed before 2009 (*n* = 1,416), 10,113 patients who were newly diagnosed between 2009 and 2018 and 101,130 age- and sex-matched controls were included in the analysis. The participants were followed-up until event occurrence, death, or December 31, 2019.

**Fig. 2:**
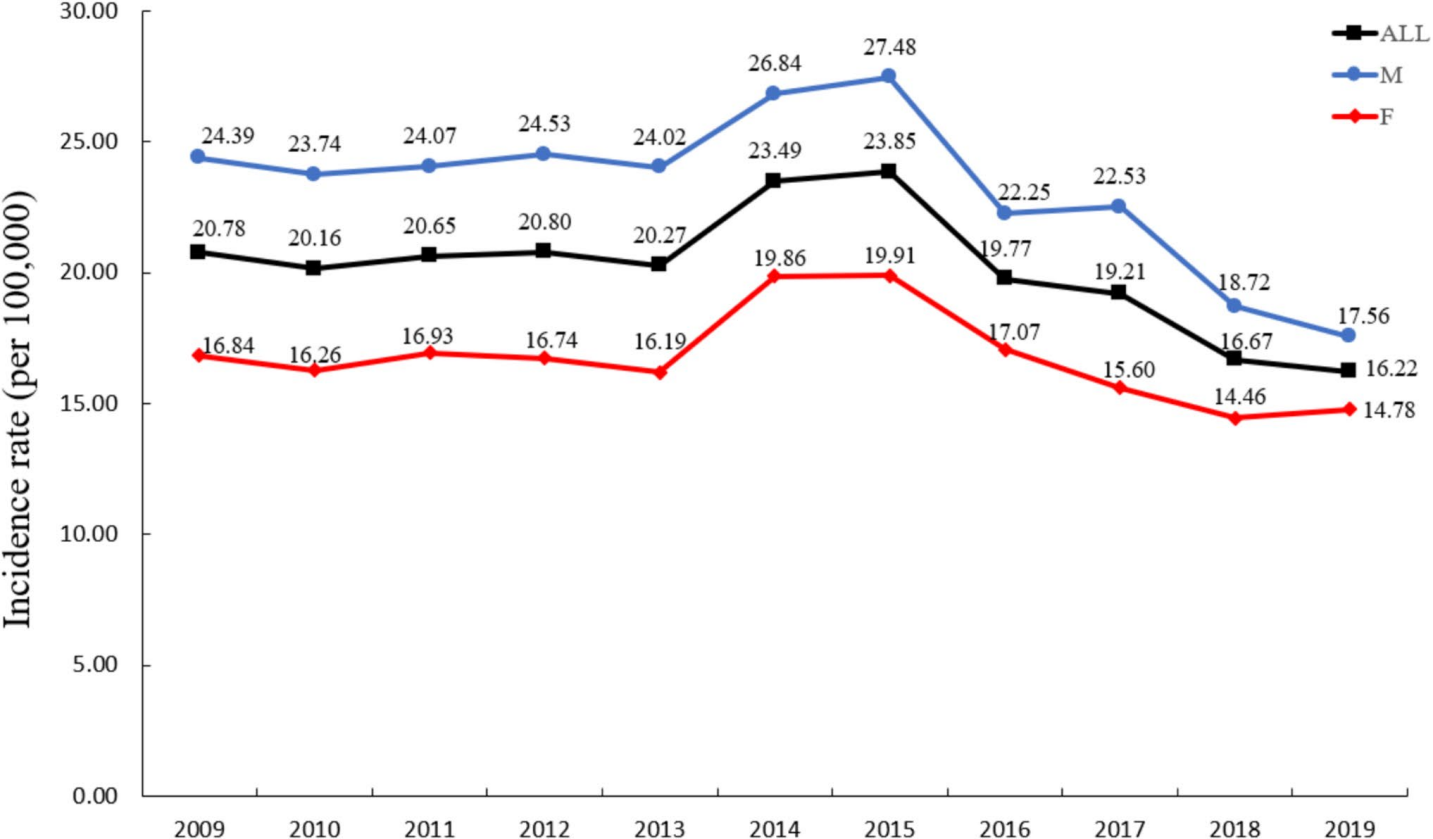
Annual incidence trends in pediatric urolithiasis in Taiwan, 2009–2019. Annual incidence rates of pediatric urolithiasis per 100,000 persons stratified by sex (male, female) and overall population. The overall incidence (black squares) declined from 20.78 per 100,000 persons in 2009 to 16.22 per 100,000 persons in 2019. Male patients (blue circles) consistently had higher incidence rates than female ones (red diamonds), with a peak rate of 27.48 per 100,000 persons in 2015, followed by a gradual decline thereafter. These findings indicate a male predominance and a modest downward trend in pediatric urolithiasis over the last decade

The annual incidence of pediatric urolithiasis declined from 23.85 to 16.22 per 100,000 persons, yielding an overall cumulative rate of 20.25 per 100,000 (Fig. 2). The mean age at diagnosis was 14.4 ± 4.4 years, and the male-to-female ratio was 1.5:1. As shown in Table 1, patients with urolithiasis had a higher exposure to several medications than controls. The most evident standardized mean differences were observed for diuretics (thiazides: 0.108, furosemide: 0.239), antimicrobials (ceftriaxone: 0.115, trimethoprim–sulfamethoxazole: 0.268, aminoglycosides: 0.273, and quinolones: 0.259), and other drugs (glucocorticoids: 0.165, magnesium trisilicate: 0.448, and nonsteroidal anti-inflammatory drugs [NSAIDs]: 0.576). The standardized mean differences for the presence of disease-related factors such as urinary tract and cyst infections were also significantly high (0.379).

**Table 1:** Baseline demographic characteristics and medication- and disease-related risk factors of pediatric patients with and without urolithiasis

	Patients with urolithiasis		Patients without urolithiasis		SMD
	(*n* = 10,113)		(*n* = 101,130)		
	*N*	%	*N*	%	
**Age (mean, SD)**	14.42	4.42	14.42	4.42	0.0000
**Sex**					
Male	6090	60.22	60,900	60.22	0.0000
Female	4023	39.78	40,230	39.78	0.0000
**Year of incidence**					
2009	1065	10.53	10,650	10.53	0.0000
2010	1005	9.94	10,050	9.94	0.0000
2011	1001	9.90	10,010	9.90	0.0000
2012	986	9.75	9860	9.75	0.0000
2013	941	9.30	9410	9.30	0.0000
2014	1062	10.50	10,620	10.50	0.0000
2015	1053	10.41	10,530	10.41	0.0000
2016	851	8.41	8510	8.41	0.0000
2017	809	8.00	8090	8.00	0.0000
2018	688	6.80	6880	6.80	0.0000
2019	652	6.45	6520	6.45	0.0000
**Medication- and disease-related risk factors of urolithiasis**					
*** Medication-related risk factors***					
**Diuretics**					
Thiazides	67	0.66	34	0.03	**0.1076**
Furosemide	321	3.17	146	0.14	**0.2392**
Potassium-sparing agents	33	0.33	127	0.13	0.0418
Combination of diuretics and potassium-sparing agents	17	0.17	8	0.01	0.0534
Acetazolamide	15	0.15	35	0.03	0.0400
**Antiepileptic drugs, AEDs**					
Topiramate	36	0.36	80	0.08	0.0598
Zonisamide	-		8	0.01	-
**Antibiotics**					
Sulfonamides	0	0.00			-
Ceftriaxone	102	1.01	146	0.14	**0.1153**
Trimoxazole	765	7.56	1932	1.91	**0.2684**
Aminoglycoside	536	5.3	695	0.69	**0.2730**
Vancomycin	42	0.42	53	0.05	0.0765
Meropenem	30	0.3	14	0.01	0.0738
Quinolones	793	7.84	2254	2.23	**0.2587**
**Antiviral drugs**					
Atazanavir	-		-		-
Acyclovir	33	0.33	201	0.2	0.0253
**Others**					
Glucocorticoids	2029	20.06	14,057	13.9	**0.1646**
Ephedrine	23	0.23	101	0.1	0.0320
Magnesium trisilicate	5882	58.16	36,764	36.35	**0.4477**
Methotrexate	18	0.18	38	0.04	0.0422
NSAIDs	8785	86.87	63,568	62.86	**0.5760**
*** Disease-related risk factors***					
** Infection**					
Urinary tract or cyst infections	822	8.13	550	0.54	**0.3794**
**Metabolic disorders**					
Hypercalciuria	38	0.38	5	0.00	0.0857
Cystinuria	-		-		-
Dyslipidemia	52	0.51	133	0.13	0.0673
Diabetes mellitus	41	0.41	143	0.14	0.0516
**Structural abnormality in the renal system**					
Congenital urinary tract anomaly	27	0.27	-		-
Cystic kidney disease	10	0.1	11	0.01	0.0384
Vesicoureteral reflux (VUR)	32	0.32	17	0.02	0.0729
Ureteropelvic junction obstruction (UPJO)	28	0.28	4	0	0.0749
** Gastrointestinal-related disorders**					
Inguinal hernia	20	0.2	64	0.06	0.0389
Inflammatory bowel syndrome	37	0.37	166	0.16	0.0409
Short-bowel/gut syndrome	0	0.00	-		-

*SMD* standardized mean difference, *SD* standard deviation, *AEDs* antiepileptic drugs, *NSAIDs* non-steroidal anti-inflammatory drug, *VUR* vesicoureteral reflux, *UPJO* ureteropelvic junction obstruction

The multivariate logistic regression analysis (Table 2) showed several independent risk factors. Among medication-related exposures, diuretics—including thiazides (aHR: 9.44), furosemide (aHR: 14.52), and combination of potassium-sparing agents (aHR: 14.26)—were strongly associated with urolithiasis. Patients who were taking antiepileptic drugs including topiramate (aHR: 2.86) and zonisamide (aHR: 3.68) were also at higher risks for urolithiasis. Antibiotics such as ceftriaxone (aHR: 2.42, *P* < 0.001), trimethoprim–sulfamethoxazole (aHR: 2.21, *P* < 0.001), aminoglycosides (aHR: 2.97, *P* < 0.001), and quinolones (aHR: 2.17, *P* < 0.001) were significantly associated with urolithiasis. The other contributors included glucocorticoids (aHR: 1.08), magnesium trisilicate (aHR: 1.63), and NSAIDs (aHR: 2.79). Among disease-related factors, hypercalciuria (aHR: 31.54), ureteropelvic junction obstruction (aHR: 19.88), congenital urinary tract anomalies (aHR: 22.58), and urinary tract infections (aHR: 8.28) showed the strongest associations with urolithiasis. Notably, hypercalciuria was rare in both groups, resulting in a small standardized mean difference at baseline; the large effect estimate should therefore be interpreted with caution, as reflected by its wide confidence interval.

**Table 2:** Medication- and disease-related risk factors of pediatric urolithiasis

Medication- and disease-related risk factors of urolithiasis								
	OR	95% CI		*P* value	aOR	95%CI		*P* value
***Medication-related risk factors***								
**Diuretics**								
Thiazides	19.83	13.12	29.98	**< 0.001**	9.44	5.89	15.14	**< 0.001**
Furosemide	22.67	18.63	27.60	**< 0.001**	14.52	11.63	18.14	**< 0.001**
Potassium-sparing agents	2.61	1.78	3.82	** < 0.001**	0.74	0.44	1.25	0.260
Combination of diuretics and potassium-sparing agents	21.45	9.24	49.77	**< 0.001**	14.26	5.53	36.78	**< 0.001**
Acetazolamide	4.29	2.34	7.86	**< 0.001**	1.93	0.88	4.22	0.100
**Antiepileptic drugs (AEDs)**								
Topiramate	4.51	3.04	6.69	**< 0.001**	2.86	1.82	4.49	**< 0.0001**
Zonisamide	5.01	1.51	16.63	**0.009**	3.68	1.01	13.38	**0.048**
** Antibiotics**								
Sulfonamides	-	-	-	-	-	-	-	-
Ceftriaxone	7.05	5.47	9.08	**< 0.001**	2.42	1.76	3.33	**< 0.001**
Trimoxazole	4.21	3.86	4.58	**< 0.001**	2.21	2.00	2.44	**< 0.001**
Aminoglycoside	8.09	7.22	9.08	** < 0.001**	2.97	2.58	3.41	**< 0.001**
Vancomycin	7.96	5.31	11.94	** < 0.001**	0.40	0.21	0.75	**0.005**
Meropenem	21.49	11.39	40.53	**< 0.001**	0.83	0.34	2.05	0.691
Quinolones	3.73	3.43	4.06	**< 0.001**	2.17	1.98	2.38	**< 0.001**
**Antiviral drugs**								
Atazanavir	-	-	-	-	-	-	-	-
Acyclovir	1.64	1.14	2.38	**0.008**	0.95	0.62	1.45	0.815
**Others**								
Glucocorticoids	1.56	1.48	1.64	**< 0.001**	1.08	1.02	1.15	**0.006**
Ephedrine	2.28	1.45	3.59	**< 0.001**	1.15	0.66	2.00	0.616
Magnesium trisilicate	2.43	2.34	2.54	**< 0.001**	1.63	1.55	1.70	**< 0.001**
Methotrexate	4.74	2.71	8.32	** < 0.001**	0.81	0.36	1.84	0.621
NSAIDs	3.91	3.69	4.15	** < 0.001**	2.79	2.62	2.97	**< 0.001**
***Disease-related risk factors***								
**Infection**								
Urinary tract or cyst infections	16.17	14.48	18.05	**< 0.001**	8.28	7.33	9.37	**< 0.001**
**Metabolic disorders**								
Hypercalciuria	76.27	30.02	193.81	**< 0.001**	31.54	11.37	87.49	**< 0.001**
Cystinuria	-	-	-	-	-	-	-	-
Dyslipidemia	3.93	2.85	5.42	**< 0.001**	2.20	1.47	3.30	**< 0.001**
Diabetes mellitus	2.88	2.03	4.07	**< 0.001**	1.52	0.99	2.36	0.059
** Structural abnormality in the renal system**								
Congenital urinary tract anomaly	67.27	23.59	191.83	**< 0.001**	22.58	6.46	78.88	**< 0.001**
Cystic kidney disease	9.10	3.86	21.43	**< 0.001**	8.34	3.25	21.35	** < 0.001**
Vesicoureteral reflux (VUR)	18.88	10.48	34.01	**< 0.001**	1.90	0.85	4.27	0.118
Ureteropelvic junction obstruction (UPJO)	69.55	24.48	197.59	** < 0.001**	19.88	5.88	67.19	**< 0.001**
**Gastrointestinal-related disorders**								
Inguinal hernia	3.13	1.90	5.18	**< 0.001**	1.86	1.03	3.36	**0.040**
Inflammatory bowel syndrome	2.23	1.56	3.19	**< 0.001**	1.68	1.15	2.45	**0.008**
Short-bowel/gut syndrome	-	-	-	-	-	-	-	-

*OR* odds ratio, *CI* confidence interval, *aOR* adjusted odds ratio, *AEDs* antiepileptic drugs, *NSAIDs* non-steroidal anti-inflammatory drug, *VUR* vesicoureteral reflux, *UPJO* ureteropelvic junction obstruction

### Complications

#### Renal complications

Children with urolithiasis experienced substantially higher rates of post-index stone-related clinical manifestations and renal outcomes compared with controls (Table 3). Hematuria, a stone-related manifestation reflecting urothelial irritation or ongoing stone activity, showed the largest increase (724.6 vs. 34.8 per 100,000 person-years; IRR: 20.84, *P* < 0.001). In terms of long-term renal complications, chronic kidney disease (13.5 vs. 2.0; IRR: 6.63, *P* < 0.001) and urinary tract/cyst infections (876.1 vs. 164.3; IRR: 5.33, *P* < 0.001) were significantly more common among stone formers. Additional renal manifestations included enuresis (IRR: 3.31, *P* < 0.001) and proteinuria (IRR: 2.20, *P* < 0.001). Multivariate analyses confirmed elevated adjusted hazard ratios for hematuria (17.76, 95% CI: 15.28–20.65), chronic kidney disease (aHR 5.92, 95% CI: 3.08–11.35), urinary tract/cyst infections (aHR 4.57, 95% CI: 4.21–4.96), enuresis (aHR 3.16), and proteinuria (aHR 2.16).

**Table 3:**
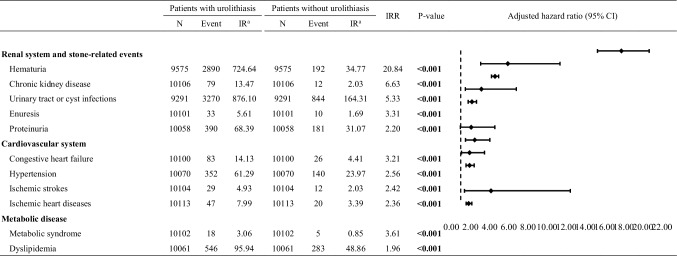
Incidence and adjusted hazard ratios of renal, cardiovascular, and metabolic complications in children with urolithiasis and matched controls

*IR* incidence rate, *IRR* incidence rate ratio

^a^IR per 100,000 person-year

#### Cardiovascular complications

Children with urolithiasis had significantly higher incidence rates of cardiovascular events. The most notable increases were for congestive heart failure (14.1 vs. 4.4, IRR: 3.21, *P* < 0.001), hypertension (61.3 vs. 24.0, IRR: 2.56, *P* < 0.001), ischemic stroke (4.9 vs. 2.0, IRR: 2.42, *P* < 0.001), and ischemic heart disease (8.0 vs. 3.4, IRR: 2.36, *P* < 0.001). The multivariate Cox models showed increased adjusted risks for congestive heart failure (aHR: 2.45, 95% CI: 1.49–4.04, *P* < 0.001), hypertension (aHR: 1.92, 95% CI: 1.11–4.90, *P* < 0.001), and ischemic heart disease (aHR: 1.93, 95% CI: 1.07–3.47, *P* = 0.03).

#### Metabolic complications

The cohort with urolithiasis presented with elevated incidence rates of metabolic syndrome (3.1 vs. 0.9 per 100,000 person-years) and dyslipidemia (95.9 vs. 48.9, *P* < 0.001). Adjusted analyses confirmed higher risks for metabolic syndrome (aHR: 4.15, 95% CI: 1.39–12.44, *P* = 0.01) and dyslipidemia (aHR: 1.83, 95% CI: 1.56–2.15, *P* < 0.001), as shown in Table 3.

### Treatment patterns

Among the 10,113 pediatric patients, 821 (8.1%) received surgical intervention. The majority of patients underwent ureteroscopy with stone removal (7.0%), followed by endoscopic cystolitholapaxy (0.9%), cystolithotomy (0.1%), and ureterolithotomy (0.08%).

Approximately 30% of patients received pharmacologic therapy. Smooth muscle relaxants—*hyoscine butylbromide* (9.5%) and *Rowapraxin* (7.1%)—were most commonly prescribed, followed by urinary alkalinizing agents such as potassium citrate (6.5%) and alpha-adrenergic blockers. Preventive medications including thiazide diuretics and uric acid-lowering agents were not frequently used. The surgical and pharmacologic management of pediatric urolithiasis is summarized in Table 4.

**Table 4:** Surgical and pharmacologic management of pediatric urolithiasis

	Patients with urolithiasis	
	(*n* = 10,113)	
	*N*	%
**Surgery**	821	8.12
**Types of surgery**		
Ureteroscopy and removal of ureteral stone with SONO/EHL	615	74.91
Ureteroscopy and removal of ureteral stone with Nd-YAG laser	58	7.06
Ureteroscopy and removal of ureteral stone—simple endoscopy	40	4.87
Ureterolithotomy—upper or distal 1/3	6	0.73
Laparoscopic ureterolithotomy	2	0.24
Cystolithotomy	12	1.46
Simple endoscopic cystolitholapaxy	25	3.05
Complex endoscopic cystolitholapaxy	63	7.67
**Drugs used**		
**Smooth muscle relaxants**		
Buscopan	960	9.49
Rowapraxin	718	7.1
** Urinary alkalinizing agents**		
Potassium citrate	657	6.5
**A-adrenergic blockers**		
Tamsulosin	379	3.75
Terazosin	24	0.24
Alfuzosin	12	0.12
Doxazosin	96	0.95
**Uric acid-reducing agents**		
Allopurinol	39	0.39
Benzbromarone	90	0.89
**Thiazides**	96	0.95

SONO/EHL, Endoscopic Ultrasonography (SONO) and Electrohydraulic Lithotripsy (EHL)

## Discussion

This nationwide study provides the largest population-based assessment of pediatric urolithiasis in Taiwan. Although its incidence has declined modestly during the past decade, affected children exhibit substantially increased risks of renal, cardiovascular, and metabolic complications. These findings underscore that pediatric urolithiasis is not merely a transient urinary condition, but a systemic disorder with long-term health implications.

The incidence of pediatric urolithiasis in Taiwan was relatively stable from 2009 to 2013, peaked in 2014–2015, and declined steadily after 2016, reaching 16.22 per 100,000 persons by the end of the study period. Although this pattern differs from the increasing or plateauing trends reported in many Western cohorts, it is consistent with earlier Taiwanese population-based studies. Prior nationwide analyses reported declining rates of newly diagnosed pediatric urolithiasis and reduced healthcare utilization from 1998 to 2007, as well as decreasing age-adjusted visit rates for upper urinary tract stones in adults between 1998 and 2010 [9, 10]. These data suggest that Taiwan may have experienced an earlier epidemiologic peak, followed by stabilization and decline.

Several factors may contribute to this trend. Improved prenatal screening has facilitated earlier detection and management of congenital urinary tract anomalies [16]. Advances in pediatric urinary tract infection management may also have contributed to reduced infection-related lithogenesis. In addition, nationwide health promotion policies targeting childhood obesity, sugar-sweetened beverage consumption, and school nutrition may have favorably influenced metabolic risk profiles, as reflected in population-based surveys such as the Nutrition and Health Survey in Taiwan (NAHSIT) 2017–2020 [17–19]. Although these factors cannot be directly measured in the NHIRD, prior Taiwanese population-based studies have proposed similar explanations for the declining incidence of pediatric urolithiasis [9, 10]. Minor variations in diagnostic recording, particularly during the ICD-9-CM to ICD-10-CM transition, may also have contributed to modest fluctuations in recorded incidence. Importantly, the observed temporal pattern remained consistent when adjacent years were examined, suggesting that the overall trend is unlikely to be driven solely by coding transitions. Taken together, the observed decline likely reflects the continuation of longer-term national epidemiologic trends combined with healthcare improvements and administrative factors.

### Etiologic heterogeneity and metabolic risk

Pediatric urolithiasis often reflects underlying metabolic disturbances, congenital anomalies, or infections rather than the lifestyle-driven mechanisms that predominate in adults [5, 20]. In our cohort, hypercalciuria emerged as the most prominent metabolic risk factor, consistent with long-standing evidence that calcium-handling abnormalities substantially increase urinary supersaturation and promote crystal nucleation and aggregation [5, 6]. These findings reinforce the central role of calcium metabolism in pediatric stone formation. Although imaging data are unavailable in the NHIRD, nephrocalcinosis-specific diagnostic codes were not included in our case definition. Hypercalciuria showed the strongest association with urolithiasis in our cohort; however, this finding should be interpreted cautiously. Despite the large adjusted hazard ratio, hypercalciuria was rare in both the urolithiasis and control groups, yielding a small standardized mean difference at baseline and a wide confidence interval around the effect estimate, indicating statistical instability rather than precise risk quantification. Nevertheless, the observed direction of association is biologically plausible and consistent with established mechanisms linking disordered calcium handling to pediatric stone formation. Thus, hypercalciuria should be regarded as a marker of metabolic susceptibility rather than a precisely quantified independent risk factor. Dyslipidemia was also associated with increased stone risk (aHR 2.2), supporting that lipid dysregulation and oxidative stress contribute to tubular injury and crystal retention [8, 21, 22]. Together, these findings highlight the need for comprehensive metabolic evaluation—including urinary calcium, serum calcium/phosphate, uric acid, and lipid profiles—even after a first stone episode in children.

### Structural and infectious contributors

Structural abnormalities such as ureteropelvic junction obstruction, vesicoureteral reflux, and cystic kidney disease further predispose to urinary stasis and infection [5]. In our analysis, congenital urinary anomalies and ureteropelvic junction obstruction increased the risk of stone formation by 22.6- and 19.9-fold, respectively. The coexistence of metabolic and structural abnormalities—as observed in up to 80% of affected children [5]—likely acts synergistically to enhance the risk of crystallization by combining urinary retention with altered solute composition. Urinary tract infection remains another important contributor: urease-producing organisms elevate urinary pH and ammonium, promoting struvite stone formation [23]. Our data showed an 8.3-fold higher risk of urolithiasis in children with a previous urinary tract infection, consistent with earlier Taiwanese reports [24, 25]. Chronic infection and inflammation may also increase reactive oxygen species, accelerating crystal nucleation and tubular injury [26]. Furthermore, systemic conditions such as inflammatory bowel disease are associated with an increased stone risk (renal stone prevalence: 6.3%) [26], caused by bile salt and fat malabsorption that alters oxalate metabolism and by reduced urinary volume and citrate levels [27]. Disease severity, surgery, and antitumor necrosis factor therapy may further compound this risk. These findings underscore the multifactorial nature of pediatric urolithiasis, in which metabolic, structural, infectious, and systemic conditions interact to promote stone formation.

### Medication-related risks and clinical stewardship

Medication exposure is another modifiable risk domain. Diuretics, antiepileptic agents, antibiotics, and NSAIDs were independently associated with the development of urolithiasis. Loop diuretics such as furosemide are associated with a 14.5-fold increased risk, likely due to calcium mobilization and hypercalciuria [28]. Thiazides—which are traditionally used to prevent recurrence—showed a paradoxical association with stone risk, possibly reflecting metabolic disturbances or confounding by indication [29]. Broad-spectrum antibiotics may alter gut microbiota, reducing *Oxalobacter formigenes* and enhancing oxalate absorption [5, 23]. NSAIDs, linked to renal papillary necrosis [30], increased urolithiasis risk 2.8-fold. Further, carbonic anhydrase inhibitors (topiramate, zonisamide) and glucocorticoids modify urinary pH, calcium, and uric acid metabolism, thereby increasing crystal formation propensity [28]. These findings emphasize that even therapeutic exposures can alter the renal milieu toward lithogenesis and should be cautiously considered in clinical practice.

### Considering these multiple risk factors, management strategies must be individualized

Most neonatal nephrocalcinosis and small stones resolve spontaneously [25]. Stone size remains a key determinant of stone management. In particular, stones measuring < 4 mm often pass spontaneously, while those measuring > 5 mm usually require intervention [15]. In our cohort, 8.12% of patients required surgery, and this value was comparable to that of a previous Taiwanese study (7.6%) [24]. Ureteroscopy was the most common procedure (86.85%), followed by endoscopic cystolitholapaxy (10.72%), cystolithotomy (1.46%), and ureterolithotomy (0.97%). Pharmacologic therapy is an important adjunct. Evidence on the use of medical expulsive therapy in children is limited [15]. However, agents such as Buscopan and Rowapraxin may relieve ureteral spasm, and tamsulosin facilitates the passage of small stones. Potassium citrate reduces recurrence by lowering urinary calcium levels and increasing pH, and uric acid-lowering agents combined with thiazides may further prevent stone formation [14]. In our cohort, the commonly used medications included Buscopan® (9.49%), Rowapraxin® (7.1%), potassium citrate (6.5%), and tamsulosin (3.75%). These findings emphasize the need for management strategies individualized based on stone size and metabolic profile, integrating pharmacologic therapy to prevent recurrence and improve outcomes.

### Systemic outcomes and clinical significance

Before addressing systemic outcomes, it is important to distinguish stone-related manifestations from long-term complications. Hematuria, which occurs in nearly 90% of pediatric urolithiasis cases, represents a stone-related clinical event rather than a systemic long-term outcome. It results from mechanical urothelial irritation and oxidative stress–related microvascular fragility [15]. In our cohort, hematuria occurred far more frequently among stone formers (IRR: 20.8; aHR: 17.8), reflecting ongoing stone activity or microlithiasis rather than systemic pathophysiology. Beyond local manifestations, our findings revealed that pediatric urolithiasis carries important systemic renal consequences. Proteinuria—an indicator of glomerular hyperfiltration or tubular dysfunction—was twice as common in stone formers (aHR: 2.16), suggesting early nephron injury [8]. Persistent proteinuria may contribute to glomerulosclerosis and tubulointerstitial fibrosis, reinforcing the chronicity of kidney damage.

Urinary tract infection also displayed a bidirectional relationship with urolithiasis: stones promote bacterial adherence and obstruction. Meanwhile, infection facilitates struvite formation and inflammation [31]. Repeated infection maintains oxidative injury, thereby promoting a cycle of inflammation and fibrosis. Thus, chronic kidney disease emerges as a major long-term outcome; stone disease accounts for up to 8% of childhood kidney failure cases [32, 33]. In our cohort, the risk of chronic kidney disease was nearly sixfold higher among stone formers. The mechanisms likely involve repeated obstruction, infection, and inflammatory insult, which reduce nephron number and renal reserve [34]. Interestingly, enuresis was more frequently observed in affected children (IRR: 3.31, aHR: 3.16), possibly reflecting impaired tubular water handling, reduced urine concentration ability, and autonomic dysregulation of bladder–sphincter coordination [35, 36].

The systemic nature of pediatric urolithiasis extends to cardiovascular and metabolic domains. Our study showed higher incidences of metabolic syndrome (IRR: 3.61, aHR: 4.15) and dyslipidemia (IRR: 1.96, aHR: 1.83) in affected children. We acknowledge that increased healthcare utilization and closer clinical surveillance among children with urolithiasis may have contributed, in part, to higher detection rates of cardiovascular and metabolic conditions. However, the persistence of these associations after adjustment for multiple comorbidities, together with biologically plausible inflammatory and metabolic pathways, suggests that surveillance bias alone is unlikely to fully explain the observed risks. This finding is similar to that of adult studies linking nephrolithiasis with insulin resistance and lipid metabolism disturbances [21]. Chronic inflammation and oxidative stress may represent the shared biological axis linking stone formation with endothelial dysfunction, reduced nitric oxide bioavailability, and vascular stiffness [37–39]. Inflammatory cytokines and activated macrophages, which are key mediators of atherosclerosis and cardiac remodeling [40, 41] may contribute to early vascular injury in this population. Consistent with adult data, our pediatric cohort exhibited two- to threefold higher risks of hypertension and ischemic heart disease [3, 8, 42]. Collectively, these findings support the concept that pediatric stone disease may be an early manifestation of systemic vascular vulnerability. The recognition of pediatric urolithiasis as a systemic metabolic–inflammatory disorder has several clinical implications. Routine follow-up of affected children should include not only imaging surveillance for recurrence but also laboratory assessment for proteinuria and dyslipidemia and blood pressure monitoring. Preventive strategies that aimed to reduce oxidative stress, improve endothelial function, and address modifiable metabolic risk factors—such as dietary habit, obesity, and physical inactivity—may attenuate long-term cardiovascular and renal complications [43].

### Strengths and limitations

The principal limitation is the reliance on administrative claims data. Although the NHIRD provides comprehensive population-wide coverage, diagnostic coding cannot verify imaging-confirmed urolithiasis, the presence of microlithiasis, or stone composition. Laboratory-based metabolic evaluations—such as urine chemistries or serum calcium and citrate levels—are also unavailable, precluding detailed characterization of metabolic phenotypes and recurrence. These constraints introduce the possibility of diagnostic misclassification, particularly for asymptomatic or incidentally detected stones. Radiologic nephrocalcinosis could not be distinguished due to lack of imaging data, as acknowledged above. Furthermore, the absence of a validation cohort from our hospital or other clinical centers limits the ability to directly assess diagnostic accuracy or case ascertainment, and the findings should therefore be interpreted with caution regarding diagnostic precision and generalizability. Genetic predispositions and unmeasured lifestyle factors may also contribute to residual confounding. In addition, temporal changes in diagnostic coding—particularly during the ICD-9-CM to ICD-10-CM transition—may have introduced minor variability in recorded incidence despite the use of strict case definitions. Future studies incorporating imaging-confirmed diagnoses and detailed biochemical phenotyping will be important to further validate these findings and clarify the underlying mechanisms linking pediatric urolithiasis to systemic outcomes.

In conclusion, the incidence of pediatric urolithiasis in Taiwan has shown a gradual decline. However, the condition remains strongly associated with substantial renal, cardiovascular, and metabolic morbidity. These findings reinforce the emerging view that urolithiasis represents a systemic disorder driven by intertwined inflammatory and metabolic pathways. Early metabolic assessment and multidisciplinary preventive care are important for preserving long-term kidney function and mitigating cardiovascular risk in this vulnerable population.

## Supplementary Information

Below is the link to the electronic supplementary material.

ESM 1 Graphical abstract(273 KB PPTX)

ESM 2 (22.5 KB DOCX)

## Data Availability

All data generated or analyzed during this study are included in this published article.

## Declarations

### Generative AI and AI-assisted technologies in the writing process

During the preparation of this work, the authors used ChatGPT (OpenAI) to assist in refining the English language and improving the clarity and logical flow of the manuscript. After using this tool, the author(s) thoroughly reviewed, revised, and verified all contents to ensure accuracy and originality, and take full responsibility for the final version of the manuscript.
