# Supplementary material for: Nationwide cohort analysis of pediatric urolithiasis: long-term metabolic, renal, and cardiovascular outcomes
Source: Pediatr Nephrol. 2026 Mar 9;41(8):2521–33. doi: 10.1007/s00467-026-07208-7 (PMC13337601; doi:10.1007/s00467-026-07208-7)
Supplement: Supplementary file 1 — Graphical abstract(273 KB PPTX) [file 467_2026_7208_MOESM1_ESM.pptx]

## Slide 1
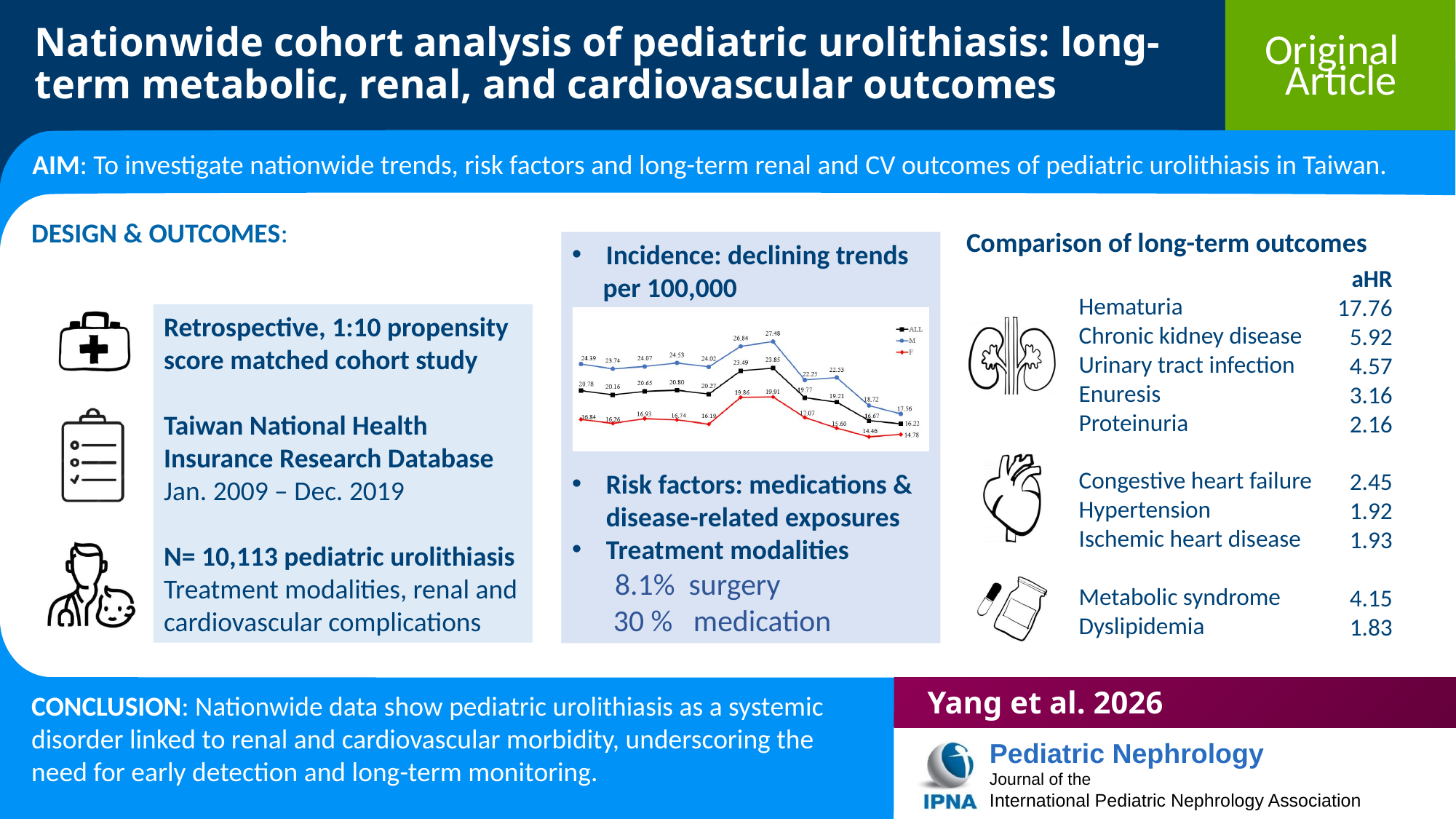

Nationwide cohort analysis of pediatric urolithiasis: long-term metabolic, renal, and cardiovascular outcomes
AIM: To investigate nationwide trends, risk factors and long-term renal and CV outcomes of pediatric urolithiasis in Taiwan.
DESIGN & OUTCOMES:
Comparison of long-term outcomes
aHR
17.76
5.92
4.57
3.16
2.16
2.45
1.92
1.93
4.15
1.83
Hematuria
Chronic kidney disease
Urinary tract infection
Enuresis
Proteinuria
Congestive heart failure
Hypertension
Ischemic heart disease
Metabolic syndrome
Dyslipidemia
Incidence: declining trends
 per 100,000
Risk factors: medications & disease-related exposures
Treatment modalities
 8.1% surgery
 30 % medication
Retrospective, 1:10 propensity score matched cohort study
Taiwan National Health
Insurance Research Database
Jan. 2009 – Dec. 2019
N= 10,113 pediatric urolithiasis
Treatment modalities, renal and cardiovascular complications
Yang et al. 2026
CONCLUSION: Nationwide data show pediatric urolithiasis as a systemic disorder linked to renal and cardiovascular morbidity, underscoring the need for early detection and long-term monitoring.
