# Supplementary material for: Nationwide cohort analysis of pediatric urolithiasis: long-term metabolic, renal, and cardiovascular outcomes
Source: Pediatr Nephrol. 2026 Mar 9;41(8):2521–33. doi: 10.1007/s00467-026-07208-7 (PMC13337601; doi:10.1007/s00467-026-07208-7)
Supplement: Supplementary file 2 — (22.5 KB DOCX) [file 467_2026_7208_MOESM2_ESM.docx]

**Supplementary Table S1.**
Diagnostic, medication, and surgical procedure codes used in this study.
This table lists the International Classification of Diseases, Ninth and Tenth Revisions (ICD-9 and ICD-10) codes used to identify diseases and comorbidities, the Anatomical Therapeutic Chemical (ATC) classification codes for medications, and the National Health Insurance order codes for urological surgical procedures.

| Diseases | ICD-9 codes | ICD-10 codes |
| --- | --- | --- |
| Urolithiasis | 592, 594, 274.11,  788.0 | N13.2, N20.0–N20.2,  N21, N23 |
| Hypercalcemia | 275.4 | E83.5 |
| Cystinuria | 270.0 | E72.01 |
| Urinary tract or cyst infections | 599.0,590.10 | N39.0, N10 |
| Congenital urinary tract anomaly | 753.9 | Q64.8, Q64.9 |
| Cystic kidney disease | 753.1 | Q61 |
| Inguinal hernia | 550 | K40 |
| Inflammatory bowel disease | 555, 556 | K50, K51 |
| Short-bowel/gut syndrome | 579.3 | K91.2, K90.8 |
| Ureteropelvic junction obstruction | 753.20,753.21 | N13.0, Q62.11, Q62.39 |
| Vesicoureteral reflux | 593.70, 593.71 | N13.7 |
| Diabetes mellitus | 250 | E08, E09, E10, E11, E12, E13 |
| Dyslipidemia | 272 | E78 |
| Proteinuria/albuminuria | 791.0 | R80.0, R80.1, R80.8, R80.9 |
| Enuresis | 788.36 | N39.44 |
| Hematuria | 599.7 | R31.0, R31.1, R31.2, R31.9 |
| Urinary tract or cyst infections | 599.0 | N39.0 |
| CKD | 585 | N18.4, N18.5, N18.6, N18.9 |
| Hypertension | 401–405 | I10, I11.0, I11.9, I12.0, I12.9, I13.0, I13.10, I13.11, I13.2, I15.0, I15.1, I15.2, I15.8, I15.9, N26.2 |
| Ischemic heart diseases | 410, 412, 414.0, 414.0, 414.2, 414.3, 414.4, 414.8, 414.9 | 121, 125.2, 125.1, 125.82, 125.83, 125.84, 125.89, 125.9 |
| Congestive heart failure | 398.91, 402.01, 402.11, 402.91, 404.01, 404.03, 404.11, 404.13,  404.91, 404.93, 425.4–425.9, 428 | I09.81, I11.0, I11.9, I13.0, I13.2, I13.10, I42.8–I42.9,  I50 |
| Ischemic strokes | 433–435 | I63, G45 |
| Metabolic syndrome | 277.9 | E88.810 |

| Drugs | ATC codes |
| --- | --- |
| Furosemide | C03CA01 |
| Thiazide | C03A |
| Potassium-sparing agents | C03D, C03E amiloride (ATC: C03DB01) or spironolactone (C03DA01) or triamterene (ATC: C03DB023) |
| Acetazolamide | S01EC016 |
| Glucocorticoids | H02AB |
| Topiramate | N03AX11 |
| Zonisamide | N03AX151 |
| Sulfonamides | J01EB |
| Ceftriaxone | J01DD04 |
| Trimoxazole | J01EE01 |
| Aminoglycoside | J01GB |
| Vancomycin | J01XA01 |
| Meropenem | J01DH02 |
| Quinolones | J01M |
| Atazanavir | J05AE081 |
| Indinavir | J05AE024 |
| Acyclovir | J05AB017 |
| Ephedrine | R03CA021 |
| Magnesium trisilicate | A02AA054 |
| Methotrexate | L01BA01,L04AX03 |
| NSAID | M01AA, M01AB, M01AC, M01AE, M01AG, M01AH, M01AX |
| Thiazides | C03A |
| Tamsulosin | G04CA02 |
| Tamsulosin | G04CA02 |
| Terazosin | G04CA03 |
| Alfuzosin | G04CA01 |
| Doxazosin | G04CA04 |
| Buscopan | A03BB01 |
| Rowapraxin | G04BD |
| Allopurinol | M04AA012 |
| Benzbromarone | M04AB03 |

Surgery codes

| Types of surgery | Order codes |
| --- | --- |
| Cystolithotomy | 78005B |
| Cystourethroscopy with removal of ureteral calculi | 78024C |
| Simple endoscopic cystolitholapaxy | 78026C |
| Complex endoscopic cystolitholapaxy | 78027C |
| Ureteroscopy and removal of ureteral stone—simple endoscopic | 77026B |
| Ureteroscopy and removal of ureteral stone with SONO/EHL | 77027B |
| Ureteroscopy and removal of ureteral stones with Nd-YAG laser | 77028B |
| Laparoscopic ureterolithotomy | 77030B |
| Ureterolithotomy—upper or distal 1/3 | 77001B |
| Ureterolithotomy—middle 1/3 | 77002B |
| Percutaneous nephrostolithotomy (PCNSL) | 76016B |
| Nephroscopy (including secondary surgical operation of PCNSL) | 76017B |
